# H1 gets the genome in shape

**DOI:** 10.1186/s13059-016-0872-9

**Published:** 2016-01-18

**Authors:** Annalisa Izzo, Robert Schneider

**Affiliations:** Institut de Génétique et de Biologie Moléculaire et Cellulaire, CNRS UMR 7104, INSERM U 964, Université de Strasbourg, 67404 Illkirch, France; Institute of Functional Epigenetics, Helmholtz Zentrum Munich, 85764 Oberschleißheim, Germany

## Abstract

By performing high-throughput chromosome conformation capture analyses in embryonic stem cells depleted of the linker histone H1, Geeven and colleagues have uncovered exciting new evidence concerning a role for this histone in modulating three-dimensional genome architecture and chromatin organization.

Please see link to Research article: http://www.genomebiology.com/2015/16/1/289

## Mapping higher-order chromatin structures

The first evidence that chromosomes are not randomly arranged in the nuclear space but that genes occupy preferential positions relative to other genomic regions and/or to nuclear structures (such as the nuclear periphery and heterochromatin foci) came from several groups in the late 1970s (for review, see [1]). However, a new era in understanding nuclear architecture has just started with the recent development of more-advanced methods to study genomic interactions in vivo. These so-called chromosome conformation capture (3C) technologies provide quantitative, high-resolution maps of the physical contacts between selected genomic regions or of those occurring genome wide [2]. These approaches led to the model that the genome is spatially arranged at several, hierarchical levels in the three-dimensional space of the cell nucleus. This organization starts with the folding of the chromatin fiber into higher-order chromatin structures, followed by the formation of loops over a wide range of genomic distances and the generation of chromatin domains referred to as topological-associated domains (TADs). It culminates in the formation of chromosome territories (CTs) [3]. The relative arrangement of TADs is largely conserved between cell types; however, TADs can undergo dynamic reorganization during differentiation [4]. The molecular mechanisms and the chromatin components responsible for the shaping of the genome and the establishment and maintenance of TADs are not yet fully understood.

An important structural component of chromatin is the linker histone H1. H1 has a well-accepted role in chromatin compaction and formation of higher-order chromatin structures in vitro. Moreover, H1 has been shown to interact with several regulatory factors and to be necessary for the recruitment of chromatin-modifying enzymes and architectural proteins [5]. A potential role for H1 in shaping the three-dimensional organization of the genome in vivo is therefore highly conceivable, but has not been addressed so far.

## Loss of H1 leads to compartmental alterations and changes in regulatory marks

**Fig. 1:**
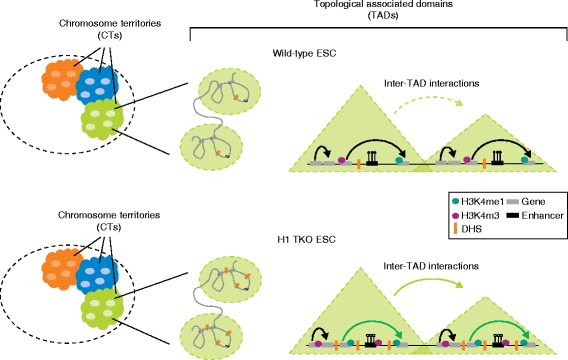
The effects of depletion of histone H1 on genome architecture. (*Top panels*) In interphase, chromosomes are organized in distinct chromatin territories (*CTs*) that are cytologically defined. Within a CT, the chromosome is structurally organized into distinct topological-associated domains (*TADs*). Within individual TADs, regulatory elements, such as enhancers and gene promoters, can be engaged in looping interactions. (*Bottom panels*) In H1 triple–knockout (*TKO*) embryonic stem cells (*ECSs*), the overall genome organization into TADs is not majorly affected. However, within gene-dense TADs, long-range inter-TAD interactions increase and new DNAse hypersensitive sites (*DHSs*) and sites of histone H3 lysine 4 mono-methylation and trimethylation (*H3K4me1* and *H3K4me3*, respectively) are established

Geeven and colleagues have investigated for the first time the potential role of H1 in genome organization in vivo [6]. They performed high-throughput chromatin conformation capture (Hi-C) analysis of the genome-wide chromatin architecture in H1 triple-knockout (TKO) mouse embryonic stem cells (mESCs). These cells harbor a deletion of three of the five replication-dependent somatic H1 subtypes (H1c, H1d and H1e), resulting in a 50 % reduction of overall H1 levels. They show that reduced amounts of H1 in mESCs cause specific changes in the structural segmentation of chromosomes, but surprisingly do not have a major effect on the overall genome organization at the three-dimensional level. This means that, although TADs are largely unaltered between wild-type and TKO cells, the frequency of their inter-domain interactions increases over long distances within single chromosome territories in the presence of limiting amounts of H1 (Fig. 1). The degree of these topological alterations correlates with the amount of changes in histone or DNA modifications occurring within individual TADs. The most profound structural changes happen within TADs where the ‘epigenetic’ landscape is extensively modified. In particular, gene-dense TADs lose DNA methylation at enhancer regions upon H1 depletion. Interestingly, only a few genomic sites gain DNA methylation in the absence of H1. CpG-rich promoters maintain their methylation status in H1 TKO cells, indicating that their methylation levels are controlled in an H1-independent manner. In H1 TKO cells new DNAse hypersensitive sites (DHSs) and new sites of H3K4me1 (a mark indicating potential enhancer elements) preferentially accumulate in gene-dense TADs. By contrast, sites losing H3K4me1 are enriched in gene-poor TADs. Surprisingly, no changes in the “repressive” histone modifications H3K9me3 and H3K27me3 levels could be detected upon H1 depletion. Thus, despite H1 being present throughout the genome, depletion of H1 results in a preferential gain of H3K4me1 and also H3K4me3 chromatin marks within the most gene-dense TADs.

By performing DamID in human cells, we have recently shown that the somatic H1 variants H1c, H1d and H1e (depleted in the H1 TKO ESCs) are relatively enriched at regions marked by repressive modifications and depleted at regions where active modifications are enriched [7]. It is therefore possible that, in the H1 TKO cells, the reduction of total H1 expression has less impact on chromatin modifications in repressive chromatin surroundings, whereas H1 levels are crucial to maintain the correct epigenetic state of active TADs, where the amount of H1 is already low in normal conditions.

One interesting observation that Geeven et al. make is that, upon H1 depletion, differentially expressed genes are randomly distributed throughout the genome and not enriched in the TADs with the highest changes of chromatin modifications and the most significant topological reorganization. Thus, chromatin modification changes are, in this case, not sufficient to predict gene expression outcomes. The authors conclude that the TADs with the most striking changes in transcriptional output are not necessarily the most sensitive to topological changes due to H1 depletion. Rather, alterations in the epigenetic landscape appear to correlate best with topological alterations of TADs.

## Pluripotency and the question of histone compensation

H1 TKO ESCs maintain their pluripotent identity as heterochromatin regions keep their unorganized spatial distribution. Also, the clustering of chromatin regions enriched in pluripotency factor genes such as *Oct4*, *Kl4* and *Sox2* is unchanged [8]. However, ESCs depleted of multiple H1 subtypes are unable to differentiate as they fail to fully repress several pluripotency genes in comparison with wild‐type cells. Extensive chromatin reorganization occurs during mESC differentiation, such as suppression of promoter–enhancer looping at pluripotency gene loci, leading to their repression [9]. In addition, pluripotent genes relocate from the nuclear center to the nuclear periphery upon differentiation of mESCs [10]. In future research, it will be interesting to investigate the effects of H1 depletion on the genome-wide interactions and nuclear repositioning of key pluripotency genes.

It will also be interesting to see how depletion of different combinations of H1 subtypes affects the three-dimensional chromatin structure and to address the role of H1 heterogeneity in genome architecture. It is important to keep in mind that depletion of H1 subtypes is generally compensated by the overexpression of the remaining subtypes. Therefore, it is possible that more dramatic topological changes in the overall chromatin organization in H1 TKO cells might be bypassed by the up regulation of the remaining subtypes (in this case for example, H1b). In our DamID analysis, we found that the subtypes H1b to H1e can be grouped together according to their genomic distribution [7] and might therefore have a common function in genome architecture. In contrast to this, the genomic localization of the H1a subtype is different; for example, it is more enriched at polycomb topological domains. It might therefore be possible that the observed effects are also a consequence of H1a upregulation [8] and not merely due to depletion of H1c, H1d and H1e.

## Concluding remarks

Together, these results provide new evidence in support of a key role for the linker histone H1—the ‘forgotten histone’—in genome architecture and chromatin organization at the three-dimensional level. What remains to be understood is how, mechanistically, H1 achieves this task and whether H1 heterogeneity plays a key role in shaping chromatin architecture at high resolution.

## Abbreviations

3C: chromosome conformation capture
CT: chromosome territory
DamID: DNA adenine methyltransferase identification
DHS: DNAse hypersensitive site
ESC: embryonic stem cell
H3K4me1: histone H3 lysine 4 mono-methylation
H3K4me3: histone H3 lysine 4 tri-methylation
Hi-C: high-throughput chromatin conformation capture
mESC: mouse embryonic stem cell
TAD: topological-associated domain
TKO: triple knockout
